# Epidermoid cyst in quadrigeminal cistern presenting with mutism

**DOI:** 10.4103/1817-1745.76120

**Published:** 2010

**Authors:** Priyanka Kawal, Raj Kumar

**Affiliations:** Department of Neurosurgery, Sanjay Gandhi Post Graduate Institute of Medical Sciences, Lucknow - 226 014, India

**Keywords:** Epidermoid, mutism, quadrigeminal cistern

## Abstract

Epidermoid cyst of the quadrigeminal cistern is uncommon, and its presentation as mutism as the main clinical finding with no other neurological finding is very rare. We report a case where the epidermoid cyst presented with progressive symptoms of absolute mutism, which improved significantly following surgery. The possible causes and pathophysiological mechanism of mutism in the lesions of this region are discussed in this paper.

## Introduction

Epidermoid cyst/tumor is a benign lesion that may arise in the spine or may arise intracranially. It may be intradural (extraaxial) or extradural (usually arising in the diplopic space of calvaria). Intracranial epidermoid cyst accounts for 1.8 -2 % of all brain tumors.[1–3] The most common intracranial location for epidermoid tumors is the cerebellopontine angle cistern, which accounts for approximately 40–50% of the cases.[4] The other locations include fourth ventricle,[5] parasellar region, intraparenchymal, the pineal gland, the thalamus and the septum pellucidum.[6] These tumors may also be seen intrinsically within the brainstem.[7] In rare cases, they have been reported in other locations such as the lateral ventricles.[8]

The term “mutism” describes patients who lack spontaneous speech despite the appearance of alertness.[9] According to Benson, there are five neurological conditions causing muteness.[10] The first is damage to the Broca’s area, which may lead to total absence of speech. The second is damage to the supplementary motor area of the dominant hemisphere and the third damage to the reticular formation of the mesencephalon, which may also leave a patient mute in the context of akinetic mustism. The fourth is pseudobulbar palsy due to diffuse bilateral cerebral hemispheric dysfunction. Muteness has also been described following bilateral thalamotomy (fifth) for Parkinson’s disease. In addition to these conditions, there are a few cases that developed transient mutism following removal of large vermian or fourth ventricle tumors.[11–17]

Mutism is more commonly related to surgery in the posterior fossa tumor. It may be immediate or delayed. Virtually all cases of mutism occur within the first week of surgery, with 50% occurring within the first 2 days. Overwhelmingly, mutism of cerebellar origin has been reported following surgical interventions in the posterior fossa for tumors. However, there are other etiologies reported in the literature, including posterior fossa trauma, cerebellitis (inflammation of the cerebellum), cerebellar hemorrhage, embolic event and arterio-venous malformations. However, epidermoid cysts of the quadrigeminal cistern presenting with mutism are not known. We report a rare case of quadrigeminal cistern epidermoid with mutism. The causes, mechanism, pathophysiology, and management of such cases are discussed here.

## Case Report

**Figure 1:**
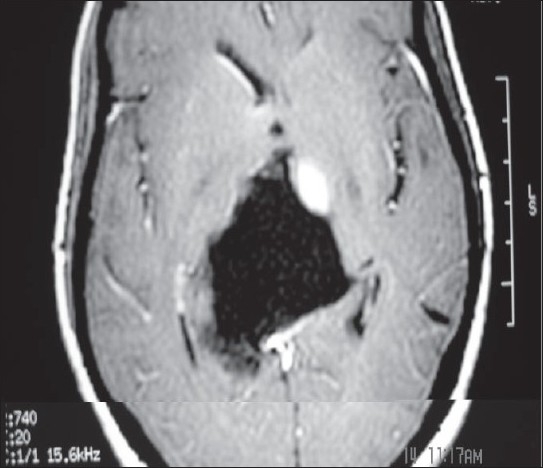
Preoperative T1-weighted axial magnetic resonance imaging showing a giant epidermoid cyst in the quadrigeminal cistern

**Figure 2:**
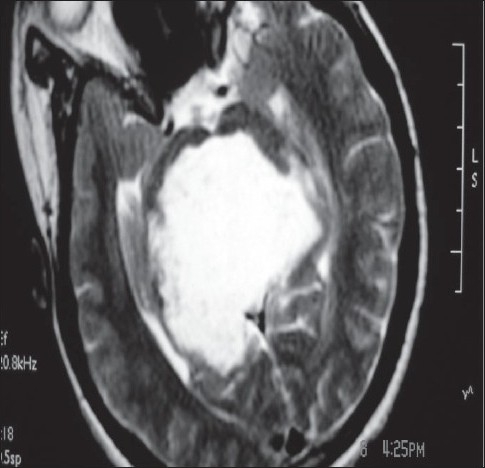
Postoperative T1-weighted axial magnetic resonance imaging showing near-total excision of the tumor; the wall is left behind

A 16-year-old female presented to us with a 10-month history of headache of mild to moderate severity. She gave history of occasional vomiting during the previous months. Her parents revealed that their daughter was failing to speak anything for the last 6 months. On examination, the child was fully conscious, following simple commands but not verbalizing at all, but she was able to gesticulate for most of the queries. She had upward gaze paresis with normal insight and reaction. Otherwise, the rest of the ocular movements remained unrestricted. Fundi showed mild papilloedema. Her routine investigations remained within normal limits. A magnetic resonance imaging image revealed a large homogeneously hypointense mass on the T1-weighted images, which was hyperintense on the T2-weighted image to occupy an entire quadrigeminal cistern [Figure 1]. It was a very large mass, expanding the cistern with extension into the supracerebellar cistern. It was almost compressing the entire third ventricle anteriorly and also the splenium as well as the posterior half of the corpus callosum superiorly. It was causing a mass effect over the upper part of the midbrain. Considering the diagnosis of quadrigeminal cistern epidermoid, the patient was planned for surgery via an infratentorial and supracerebellar approach. During surgery, the epidermoid was encountered in the supracerebellar cistern itself. The epidermoid was encircling the vessels of the cistern, i.e. vein of Galen, basal veins of Rosenthal and posterior part of the internal cerebral veins. The entire pearly material of the epidermoid was delivered from all the sites gradually, leaving the thin capsule behind at places [Figure 2]. A large cavity was created and cerebrospinal fluid (CSF) started pouring in from the posterior third ventricle. Once the quadrigeminal cistern became clear, a ventricular catheter was left in the cavity and the wound was closed as per the standard norms. The patient was extubated following surgery. It was very surprising to note that the patient started verbalizing immediately after complete recovery from anesthesia. The ventricular drain was taken out after 24 h as the CSF drained was clear. She made an uneventful recovery without any neurological deficit. She was discharged on the 7^th^ postoperative day. The patient was continued on steroids for the next 2 weeks in order to take care of chemical meningitis. At the follow-up of 6 months, the girl was perfectly well.

## Discussion

There are numerous case reports of mutism in the literature. In a report, a 14-year-old girl with epidermoid cyst in the 3^rd^ ventricle manifested mutism. The cyst was tapped three times and, after each tapping, the patient improved, but after a few days the symptoms recurred.[18] Therefore, excision of cyst was performed. Postoperatively, in the first week, her speech was mainly limited to yes or no. On discharge from the hospital after 8 weeks of operation, she was still drowsy. At the 8-month follow-up, there was no mutism, but she was forgetful, and at times incontinent to urine. Meanwhile, during the course of her illness, there were symptoms of raised intracranial pressure (ICP). Her symptoms could be related to the direct pressure on the diencephalon level, with involvement of the hypothalamic–thalamic communication, with impairment of the afferent impulse. In our case, the cyst was located in the quadrigeminal cistern, and improvement was dramatic. This signifies the importance of some anatomical substrate responsible for mutism in close vicinity of the quadrigeminal cistern.

Transient mutism has been reported in three cases following removal of the lateral and third ventricular mass following a transcallosal approach.[19] Mutism may be a result of division of the corpus callosum in such cases. Suppression of the limbic system caused by a lesion in the anterior cingulate gyrus, septum pellucidum, fornix, supplementary motor cortex, thalamus and basal ganglion may be responsible for reduced speech production.

Transient mutism had also been reported in a 65-year-old man following resection of anterior falx meningioma; the lesion being at the supplementary motor area may be responsible for postoperative mutism in this area.[20] A 13-year-old girl with exophytic pontine glioma developed mutism after total excision of tumor, which resolved after 6 months.[21] Guidetti observed total inability to speak in two patients in whom simultaneous and bilateral lesions of the dentate nuclei were stereotactically created in order to treat spasticity.[22] According to Robertson, cerebellar mutism syndrome occurred in 107 (24%) of 450 children with medulloblastoma. The symptom intensity was judged to be severe in 43%, moderate in 49% and mild in 8% of 107 patients. Mutism and ataxia were the features most frequently judged as severe. In both cohorts, preoperative brainstem invasion was the only feature that correlated with risk of mutism.[23]

Jonathan reported two patients, each of whom developed cerebellar mutism after tumor resection, using a posterior fossa approach. The first patient underwent gross total resection of a pineal region tumor via a supracerebellar approach.[24] The second patient underwent posterior fossa decompression for a left cerebellar hemispheric renal cell carcinoma metastasis with adjacent hemorrhage.[25] One patient displayed a variant of cerebellar mutism with severe ataxic dysarthria without complete absence of speech, whereas the other demonstrated frank mutism. After neuroimaging studies confirmed the absence of a surgically treatable postoperative cause for the patients’ symptoms, they were managed in a supportive fashion (e.g., speech therapy) and improved within 3.5 months and 1 year, respectively.

Forty-six cases of cerebellar mutism with mean age ±10.4 years following posterior cranial fossa surgery were studied. The pathological lesions were medulloblastomas in 33, astrocytomas in seven, ependymomas in four, metastases tumor in one and arteriovenous malformation in one. All mass lesions were considered to be large or very large.[26] The latency for the development of mutism in these cases lasted from 4 days to 4 months (mean 6.8 weeks). Dysarthric speech ensued after the mutism was resolved in 35 of 46 patients. The mutism was transient in all the cases.

It is apparent from all the above reports that neither the occurrence of mutism was a sole manifestation in different cases nor the improvement was very dramatic following surgery. But, in our case report, mutism was the only presenting complaint with epidermoid cyst in the quadrigeminal cistern, and its improvement immediately after surgery denotes that some anatomical structure in this region is directly responsible for such a severe mutism, if compromised significantly. Although this epidermoid was not confined only to the quadrigeminal cistern, it was sizeable enough to cause a significant compression over the surrounding structures. It denotes that either one anatomical structure or bilateral involvement of one or more than one structure was responsible for such a severe mutism. The mutism in our case could be due to pressure at the reticular formation of the mesencephalon, posterior corpus callosum, bilateral fornices, bilateral thalami or a combination of all these structures.[27]

Mutism is a well known event after surgical intervention of tumors of the posterior fossa. This event is thought to be more evident in cases of tumors in which the brain stem is in some way mistreated during surgery, but it is clear that this type of mutism is speech disturbance, a motor disorder in which the cerebellum temporarily loses its capacity to poke the phonic nuclei function efficaciously to the point of anorthia and mutism.[28] However, there are other types of mutism, which are language alterations secondary to cerebellum lesions. It seems probable that cerebellar mutism (secondary to posterior fossa involvement) is relatively different in relation to supratentorial mutism. The structures involved in both of these may vary.

## Conclusion

It seems probable that the anatomical substrate for mutism lies in close vicinity to the quadrigeminal cistern, which may be either reticular formation of midbrain, fornices, thalami or corpus callosum. However, the possibility of bilateral involvement of these paired anatomical structures cannot be denied. Compression of the midbrain may also be responsible for such an event.
